# Research Progress of Micro-Nano Bubbles (MNBs) in Petroleum Engineering

**DOI:** 10.3390/gels11110866

**Published:** 2025-10-29

**Authors:** Yubo Lan, Dongyan Qi, Jiawei Li, Tong Yu, Tianyang Liu, Wenting Guan, Min Yuan, Kunpeng Wan, Zhengxiao Xu

**Affiliations:** 1 State Key Laboratory of Continental Shale Oil, Daqing 163712, China; 2 Department of Informatization and Cyber Security, Shanghai Police College, Shanghai 200137, China; 3 School of Petroleum and Natural Gas Engineering, Changzhou University, Changzhou 213164, China

**Keywords:** micro-nano bubble, physicochemical properties, gel, petroleum engineering

## Abstract

Micro-nano bubbles (MNBs), typically characterized by diameters ranging from tens of micrometers to hundreds of nanometers, have gained significant attention in recent years due to advancements in nanotechnology and related characterization methods. This technology has shown great promise in the field of petroleum engineering. Among the various applications, the integration of MNBs with gel technology plays a critical role in enhancing drilling safety. This paper aims to systematically review the current status, challenges, and optimization strategies for the application of MNBs in petroleum engineering, with a particular focus on their combined use with gel technology in oilfield applications. The paper first introduces the preparation methods and physicochemical properties of MNBs tailored for oilfield applications. It then systematically reviews the use of MNBs in the following three key areas of petroleum engineering: drilling, enhanced oil recovery (EOR), and oil–water separation. The paper also compares domestic and international technological approaches, highlighting the challenges associated with the large-scale application of MNBs in China. Notably, in the areas of drilling and enhanced oil recovery, the synergistic use of MNBs and gel technology has demonstrated significant potential. The gel–MNB combined technology demonstrates particular promise for China’s special reservoirs, as gel’s high molecular weight compensates for MNBs’ sedimentation defects, while their synergistic effects on interfacial tension reduction and drilling fluid stabilization provide an eco-efficient approach for extreme conditions. Additionally, focusing on the combined application of gel and MNB technology, along with adjustments in gel stability and MNB size, could offer a promising solution for the efficient and sustainable development of special reservoirs (such as those with high temperature, pressure, and salinity) in China.

## 1. Introduction

The International Organization for Standardization (ISO) defines bubbles with a diameter smaller than 100 μm as microbubbles. Among these, micro-nano bubbles (micro-nano bubble, abbreviated as MNBs) typically refer to bubbles with diameters ranging from tens of micrometers to hundreds of nanometers [1,2,3]. Distinct from traditional bubbles, which are typically millimeter-sized or larger, MNBs exhibit unique physicochemical properties. These properties include high stability, slow ascent rates, a significant interfacial potential, and strong oxidative characteristics. In recent years, MNBs have been extensively researched and applied in various fields, including environmental and ecological engineering, biomedicine, energy and chemical engineering, and food engineering [4,5,6]. There have been continuous advancements and breakthroughs in their physicochemical properties and end-use applications.

Petroleum engineering is a crucial element of the global energy sector. As oil reserves gradually diminish, the technological requirements in fields such as oil extraction, drilling, and oil–gas separation are increasing [7,8,9]. The application of micro-nano bubble technology in petroleum engineering has evolved from initial exploration to rapid development in recent years. As early as the 1970s, Bowonder et al. observed and analyzed the generation and phenomena of microbubbles (MBs) [1]. At the beginning of the 20th century, scholars such as Masayoshi T investigated the stability of micro-nano bubbles and the effects of surfactants. Their findings revealed the dispersibility and long lifespan of these bubbles in liquids, thereby laying the foundation for subsequent research [10]. Later, Etchepare R et al. investigated the application of nanobubbles in the separation of oil and water, revealing that microbubbles could substantially reduce the oil–water interfacial tension, thereby improving the efficiency of the separation process [11]. These early studies established a theoretical foundation for the subsequent advancement of micro-nano bubble technology in petroleum engineering, particularly in the optimization of drilling fluids [12,13] and improving oil–gas recovery rates [12,14]. In recent years, research on the application of micro-nano bubble technology in petroleum engineering has deepened. Koirala, N. investigated the role of microbubbles in oil–water separation, suggesting that the injection of microbubbles could enhance the buoyancy of oil droplets, thereby improving the efficiency of separation [15]. Concurrently, with the rise in unconventional oil and gas development, researchers such as Zhao have applied microbubble technology to enhance oil recovery EOR [16]. Their findings demonstrate that microbubbles effectively alter fluid behavior in reservoirs, reduce oil–water interfacial tension, and exhibit promising effects on production enhancement, particularly in low-permeability reservoirs [16]. Recent studies have extended beyond the laboratory phase, focusing on industrial-scale applications. Cheng et al. experimentally validated the application of microbubble technology in the treatment of oilfield wastewater, demonstrating its effectiveness in enhancing oil–water separation efficiency under actual industrial conditions, while also reducing environmental pollution [17]. In recent years, advancements in bubble miniaturization research [17,18] and the establishment of international standards have significantly broadened the application of micro-nano bubble technology in petroleum engineering [19,20,21].

This paper provides an overview of the preparation methods and characteristics of MNBs, with a focus on summarizing their application methods and processes in the field of petroleum engineering. This article examines the application of micro-nano bubble technology in petroleum engineering from three perspectives: the current application status, technical challenges both domestically and internationally, and the challenges associated with large-scale implementation in China. Each section aims to provide a comprehensive overview of the advancements and limitations of this technology within the field. This study systematically correlates bubble dynamics with the geological conditions of oil and gas reservoirs for the first time, elucidating the representativeness of three major application areas. Finally, optimization pathways for micro-nano bubble technology are proposed for Chinese unique oil and gas reservoir conditions, offering suggestions to overcome technical application challenges.

## 2. Results and Discussion

### 2.1. Micro-Nano Bubble Generation Technologies

#### 2.1.1. Micro-Nano Bubble Preparation Technology

Current techniques for preparing micro-nano bubbles primarily include seven methods such as dissolved gas, ultrasonic, and Venturi methods [22]. Table 1 summarizes the fundamental principles, technical advantages, and limitations of each approach.

As shown in Table 2, although the dissolved gas method has a high gas dissolution capacity [23], its high energy consumption limits its industrial application. The size controllability of the ultrasonic method (±50 nm) is consistent with the oil–water separation study by Etchepare et al., but local effect variations (such as temperature fluctuations of ±5 °C) may lead to batch differences [11].

The primary issues with current micro-nano bubble generators include high energy consumption of the equipment, poor uniformity of the prepared dispersion system, and low efficiency and stability of the equipment. Research indicates that utilizing surfactants as the foaming base liquid significantly enhances both the foaming quality and the bubble stability of the device. Reducing interfacial tension and improving bubble interface characteristics are considered the key mechanisms behind this effect [24]. N. Nirmalkar and A. W generated bulk nano bubbles in pure water using a high-pressure microfluidic device. The study highlights that the incorporation of nonionic surfactants imparts spatial stability to the suspension [25]. Furthermore, an increase in the concentration of anionic surfactants enhances the surface charge, thereby improving the stability of the nano bubbles. In contrast, cationic surfactants gradually neutralize the surface potential, resulting in charge reversal at the interface of the nano bubbles, which subsequently leads to a reversal in the stability of the suspension [26]. Therefore, surfactants are of great significance for enhancing bubble stability and their adaptability to the environment.

#### 2.1.2. Preparation Technology Combining Gel and Micro-Nano Bubbles

The preparation of gel foam typically involves a combination of gelling agents and cross-linking agents. For instance, polyvinyl alcohol as a gelling agent, when combined with an organic boron cross-linker, can yield a highly efficient gel foam system [27]. This gel foam not only exhibits the characteristics of non-Newtonian fluids but also demonstrates thixotropic behavior, effectively extending vapor suppression time [27,28]. In the field of petroleum engineering, the preparation of gel foam also involves the application of micro-foam drilling fluids. By adjusting the density and stability of micro-foam drilling fluids, a retention layer similar to a “liquid casing” can be formed, thereby preventing fluid loss during drilling operations [29].

Although gel micro-nano bubbles cause less environmental pollution, the chemical additives in their components may pose potential risks to groundwater and the ecological environment, especially when used for a long time [30].

### 2.2. Physical and Chemical Properties of Micro-Nano Bubbles

MNBs possess an extremely high specific surface area, making surface tension the dominant factor governing bubble behavior. According to Laplace’s law, there exists a significant pressure difference between the interior and exterior of MNBs, characterized by a substantially higher internal pressure compared to the external pressure. This enables the bubbles to maintain high gas dissolution capacity and strong surface reactivity [31]. As the size of bubbles decreases, both their surface energy and interfacial activity increase, establishing a strong foundation for their application in petroleum engineering. Due to their extremely small size, MNBs rapidly alter the interfacial tension in liquids and interact intensely with oils, gases, or solid particles present within the liquid [11,32]. MNBs exhibit strong lipophilicity and a high specific surface area, which grants them unique advantages in oil–water separation, pollutant removal, and gas dissolution. These properties highlight the immense potential of MNBs in petroleum engineering applications.

#### 2.2.1. Internal High Pressure

The high pressure within MNBs is primarily attributed to the significant curvature of the bubbles. At the micro-nano scale, this curvature becomes exceedingly pronounced. According to classical thermodynamic calculations based on the Young–Laplace equation, the relationship between the bubble diameter and the pressure within the gas cavity is described by Equation (1) [11]:

(1) $$P_{\mathrm{in}}\;=\;P_{\mathrm{out}}+\frac{2\gamma }{r}$$

where *P*_in_ is the internal pressure in the bubble; *P*_out_ is the pressure of bulk liquid; γ is the surface tension of liquid; r is the radius of the bubble. According to Equation (1), assuming r = 200 nm, *P*_out_ = 105 N/m^2^, γ = 72 mN/m, the pressure inside the bubble is 8.20 × 10^5^ (N/m^2^), which is approximately 8 times the atmospheric pressure. Therefore, the internal pressure of MNBs is significantly higher than the external pressure of the bulk solution.

**Figure 1:**
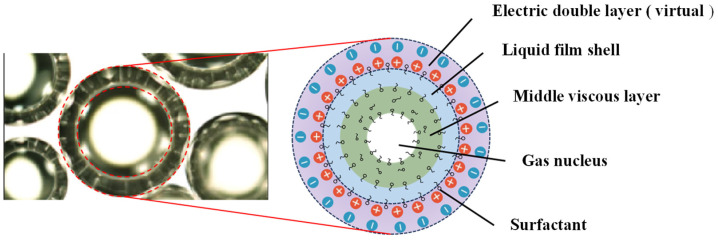
Schematic diagram of the basic structure of MNBs.

As shown in Figure 1, the multi-layer structure of micro-nano bubbles (including a gas core, a viscous intermediate layer, and a surfactant shell) is the key physical basis for their ability to maintain high pressure (calculated values up to 8 times atmospheric pressure). This layered structure stabilizes the internal pressure of the bubbles through the synergistic effect of interfacial tension (γ) and radius of curvature (r).

The high pressure inside the bubble (8.20 × 10^5^ N/m^2^) significantly inhibits its Brownian motion, thereby prolonging the suspension time [24]. According to the research of Xiao, the impact factor caused by surface contaminants inhibiting internal gas circulation ranges from 1.2 to 1.5. Surface contaminants can further reduce the rising speed and enhance the suspension stability in the drilling fluid by inhibiting the internal gas circulation [24].

Due to the extremely small radius of MNBs (typically on the micron or nanometer scale), the pressure difference is very large. The high pressure within the bubble induces dynamic behaviors, including bubble rupture and interactions between the bubble and the surrounding liquid. Additionally, the stability of MNBs is influenced by various external factors, including dissolved gases, temperature, and the type of liquid. These factors have the ability to significantly affect the internal pressure and volume of the bubbles.

#### 2.2.2. Large Specific Surface Area

MBNs have an approximately spherical structure. Stephen Brunauer first proposed the calculation formula for the specific surface area (SSA) of dispersed spherical MBNs in 1938, namely [11,32]:

(2) $$\mathrm{SSA}=\frac{6\sum n_{i}d_{i}^{2}}{\sum n_{i}d_{i}^{3}}$$

where *n_i_* represents the number of *i*th size levels, *d_i_* represents the diameter of the sphere of the *i*th size grade.

According to Equation (2), the SSA of MNBs exhibits an inverse proportional relationship with diameter. Under equivalent volume conditions, the SSA of MNBs with an average diameter of 200 nm is 10,000 times greater than that of bubbles with an average diameter of 2 mm. The significantly higher SSA of MBNs facilitates a larger gas–liquid interface, which is a critical prerequisite for enhancing flotation performance, oxidation capability, and mass transfer efficiency.

#### 2.2.3. High ζ Potential

As a dispersion system in aqueous solutions, MBNs typically exhibit a negative charge at their interface. This phenomenon arises from the dipole moment of water molecules and the adsorption of anions at the gas–liquid interface [33,34]. According to the principle of Coulombic interaction, these negative charges at the gas–liquid interface attract a surrounding counterion layer, thereby forming the extended electrical double layer (EDL) of MBNs [35]. (Fernanda Yumi Ushikubo) measured the absolute potential ranges of MBNs for various gases in water: 34–45 mV (oxygen), 1720 mV (air), 29–35 mV (nitrogen), 20–27 mV (carbon dioxide), and 11–22 mV (xenon) [26]. (N. Nirmalkar) proposed that the stability of nano bubbles is attributed to the external electrostatic pressure exerted by surface charges, which balances the internal Laplace pressure [36].

#### 2.2.4. Slow Rate of Rise

The ascent of bubbles within the bulk solution is a common phenomenon. Typically, bubbles that are millimeter-sized or larger ascend rapidly to the liquid surface, where they subsequently burst. However, extensive observational phenomena and theoretical studies indicate that the ascent velocity of MBNs in liquid is significantly slower than that of millimeter-sized and larger bubbles [37].

The inertial force of MBNs in viscous laminar flow is significantly smaller than the forces exerted by surface tension or viscosity, rendering it negligible. However, as an unavoidable factor in practical observations and calculations, the internal gas circulation within bubbles does influence their mobility to a certain extent. Consequently, suppressing the internal motion of bubbles increases the frictional force exerted by the liquid on the bubbles. Marwa Sakr defined this influence as the factor (*f*_sc_), which is caused by the inhibition of internal gas circulation due to surface contaminants [38]. Consequently, in fluid mechanics, the rising speed of small bubbles is describable by Equation (3).

(3) $$v_{b,\mathrm{vis}}=\frac{1}{f_{\mathrm{sc}}}\cdot \frac{g\rho_{L}d_{e}^{2}}{12\mu_{L}}$$

where *v*_b,vis_ represents the bubble rise velocity, g is the gravitational acceleration, *ρ*_L_ denotes the density of the liquid medium, *d*_e_ is the equivalent volume diameter of the bubble, and *μ*_L_ signifies the viscosity of the liquid medium. In reality, the value of *f*_sc_ varies between 1 and 1.5, depending on the specific contaminants present and their concentration. When the bubbles are very small and contaminated, thus preventing internal circulation, the value of *f*_sc_ is 1.5, whereas for uncontaminated or sufficiently large bubbles where internal circulation is fully developed, the value of *f*_sc_ is 1 [39]. From Equation (1), the bubble diameter, liquid viscosity, and bubble interfacial properties are key factors influencing the rise velocity. Additionally, some scholars have analyzed the reasons for the slow rise velocity of MBNs based on the principles of buoyancy calculations [35].

#### 2.2.5. High Stability

The stability mechanism of MNBs remains unclear. Numerous theories and models have been proposed to elucidate the exceptionally long lifespan of MNBs, including surface charge theory, pollution adsorption, and interfacial effects.

Some viewpoints suggest that surface tension is the primary reason for the stability of MNBs. Due to their small size and high surface tension, MNBs typically exhibit strong surface energy, which enables them to maintain stability for extended periods under specific conditions. The stability of MNBs is influenced not only by their size but also by the substances present on their surfaces, such as surfactants [17]. Other perspectives suggest that the self-organizing behavior of bubbles plays a significant role in their inclination towards a stable state. The distribution and morphology of MNBs in liquid are influenced by hydrodynamic conditions, including shear forces and buoyancy, which may lead to specific self-organizing behaviors. Additionally, the components at the bubble interface (e.g., lipids) achieve assembly functions, a behavior critical to bubble stability [11,37].

The extended DLVO theory is utilized to mechanically elucidate the stability of bubble interfaces, suggesting that the stability of MNBs relies on the equilibrium among electrostatic forces, van der Waals forces, and hydrophobic interactions. Extensive studies have demonstrated that MNBs are able to persist in bulk solutions for several months; however, the mechanisms underlying their stabilization require further refinement [1,2,3,7,26].

#### 2.2.6. High Mass Transfer Efficiency

The high mass transfer efficiency of MNBs is manifested in the generation of dissolved gases and free radicals through the gas–liquid interface, as well as the enhancement of the mass transfer process through energy release via bubble collapse. When MNBs form, the gas–liquid boundary layer possesses high molecular potential energy, driving the molecules at the boundary layer to move toward the liquid interior, thereby stabilizing the bubbles. Macroscopically, this appears as bubble dissolution and surface area reduction. The spherical interface is continuously compressed under surface tension, allowing more gas to dissolve into the bulk solution, thereby producing dissolved gases, which further induce the formation of substances such as free radicals and reactive oxygen species.

Notably, the bursting of MBNs refers to the abrupt rupture of MNBs when they contract in water to a certain extent, leading to a drastic disappearance of the gas–liquid interface. During the bursting moment, the high concentration of ions accumulated at the gas–liquid interface releases stored chemical energy instantaneously, generating a large number of free radicals [38].

### 2.3. The Stability and Mechanism of Gel Micro-Nano Bubbles

The stability of MNBs is the foundation of their application. Research indicates that the destabilization forms of MNBs primarily include drainage, sedimentation, or the coexistence of both. Factors influencing MNBs stability include the viscosity of the base fluid, shear force, molecular weight of the foam stabilizer, stirring speed, temperature, and the concentration of metal ions (such as Na^+^ and Ca^2+^) [40]. For instance, when the base fluid viscosity is low or the foam-stabilizing medium is a lightweight gel, MNBs are prone to drainage, whereas when the foam-stabilizing medium has high density or the gel molecular weight is excessively large, sedimentation is more likely to occur [40]. Additionally, the introduction of silica gel and lamellar liquid crystal gel can significantly enhance MNBs’ stability. Silica gel, by forming spherical bubbles and encapsulating them with a gel layer, enables MNBs to remain stable for over 48 h [41], while lamellar liquid crystal gel, by strengthening the liquid film and slowing the gas-phase diffusion rate, extends MNBs’ stability to as long as 10 months [42].

With the increase in NaCl concentration, the half-life of the microfoam system first decreases and then increases, and the density rises slowly [40]. Relevant researchers have found that the form of instability is manifested as sedimentation first. As the concentration of NaCl increases to 15%, it then shows as liquid separation [40]. A large amount of Na^+^ penetrates into the gel structure of the base solution, which causes the gel to have less force in binding free water. A large amount of liquid phase precipitates with the dissolved Na^+^, resulting in a dense liquid phase [40]. The large density causes it to sink, while the upper layer of microbubbles has a light density and floats up [40]. Therefore, when preparing gel microbubbles, attention should be paid to the concentration of metal ions in the application environment.

## 3. Application Example

### 3.1. Preparation of Micro-Nano Bubble Dispersion System Suitable for High-Temperature, High-Pressure and High-Salt Environment

Adaptability challenges under high-temperature, high-pressure, and high-salinity conditions in oilfields: Typically, elevated temperatures cause bubbles to rupture or dissolve, particularly in drilling and stimulation fluids, which significantly reduces the lifespan of micro-nano bubbles. This not only affects their capacity to carry cuttings during oil and gas extraction but also diminishes their role in enhancing fluid mobility and improving recovery rates in oilfields. The rheological changes in drilling fluids at elevated temperatures may interact adversely with the effects introduced by micro-nano bubbles, resulting in a deterioration of fluid performance. The high-pressure environment deep within reservoirs also poses difficulties for the application of micro-nano bubble. Under such high pressure, bubbles undergo compression, which reduces their volume and subsequently diminishes both their generation and carrying capacity. While micro-nano bubbles generally exhibit favorable performance under conventional pressures, their size and distribution become increasingly challenging to control in high-pressure environments, leading to suboptimal performance outcomes. Certain oil and gas fields in China, particularly those located in the northwest and offshore regions, encounter high-salinity conditions. The presence of water in these high-salinity environments modifies surface tension, which in turn influences the generation and stability of micro-nano bubbles. In environments with elevated salt concentrations, the generation and maintenance of micro-nano bubbles become increasingly challenging, rendering the technology less effective in comparison to low-salinity conditions.

Optimization path: On one hand, to enhance the stability of bubbles in high-temperature and high-pressure environments, it is necessary to select a bubble system adapted to such conditions. Taking Fiber Pouch Fluid as an example, which contains numerous heterogeneous bubbles dispersed in the fluid—often referred to as “recyclable foam fluid” or “microbubble fluid” in field applications [43]—the oil-displacement mechanisms of Fiber Pouch Fluid primarily consist of four types: the emulsification effect of the fiber pouch base fluid, the extrusion-carrying mechanism of the fiber pouch structure, the wedging mechanism of the fibrous structure, and the entrainment carrying mechanism. In other words, the base fluid and the foam it forms are crucial for enhancing recovery rates, and the same applies to micro-nano bubbles [44]. Thus, a stable micro-nano bubble system must not only exhibit high-temperature resistance but also possess excellent dispersion properties to ensure uniform bubble distribution. On the other hand, by modifying the surface coating of micro-nano bubbles, their pressure and heat resistance can be improved. High-temperature-resistant materials, such as nanoparticles or polymers, can be used as surface coatings to enhance bubble stability. Additionally, optimizing bubble generation techniques is essential to maintain stable micro-nano bubbles under extreme conditions. For instance, employing bubble nucleation technology to control bubble size and generation rates ensures that bubbles remain dispersed in high-temperature and high-pressure environments [45].

### 3.2. Optimization of Micro-Nano Bubble Size and Stability Based on Fracture–Pore Parameter Matching

Adaptability challenges of micro-nano bubbles in relation to China’s complex oil and gas reservoir types: In western China, particularly in the Tarim Basin, carbonate reservoirs typically demonstrate considerable variations in porosity and permeability. These reservoirs are characterized by various structural features, including faults and folds. In high-porosity reservoirs, particularly those that are fractured, micro-nano bubbles may rapidly infiltrate large pores and dissipate, significantly influencing oil and gas extraction. In recent years, the development potential of tight oil and gas reservoirs in China has garnered significant attention. Low-permeability reservoirs often contain numerous micro-fractures or nano-scale pores that hinder the penetration of bubbles into the reservoir. This results in an uneven distribution of bubbles, which subsequently hinders enhanced recovery rates. Many oil and gas reservoirs are characterized by complex geological structures. Under multi-scale fracture–pore conditions, tracking the migration behavior of micro-nano bubbles (MNBs) presents significant challenges. These challenges encompass complex bubble dynamics, irregular pore structures, strong interactions between bubbles and solid surfaces, and inadequate measurement accuracy. Such factors complicate the precise prediction of bubble movement paths and their distribution in intricate geological settings [2].

Optimization pathway: The effectiveness of micro-nano bubble technology is significantly influenced by its propagation capabilities within porous media, such as the rocks present in oil and gas reservoirs. Therefore, the size distribution of the bubbles must align with the pore-permeability conditions of the medium, particularly in heterogeneous formations where MNBs with specific particle gradations are essential for achieving optimal oil and gas displacement efficiency.

#### 3.2.1. Bubble Size Matching

First, based on the pore size distribution characteristics of the reservoir, it is essential to design an appropriate range for bubble sizes. For instance, in the case of fluffy capsule fluid, the size of the bubbles significantly influences both the fluid’s injection performance and its capacity to plug the pores effectively [46]. In the selection of microbubbles and nano bubbles, it is essential to choose larger bubbles for reservoirs that contain larger pores, whereas smaller bubbles are more appropriate for reservoirs with smaller pores. Simultaneously, adaptability testing is required, that is, experimental research and field tests should be conducted to determine the permeability, stability, and adsorption capacity of different-sized bubbles in various types of reservoirs, thereby optimizing the matching of bubble sizes.

Nonionic surfactants (such as Ween −80) significantly extend bubble lifetime through multiple mechanisms, including enhancing ζ potential and inhibiting Ostwald maturation. The ζ potential is enhanced by increasing the charge density on the surface of the bubble. The increase in ζ potential means that the electrostatic repulsive force between bubbles is enhanced, thereby inhibiting the coalescence and rupture of bubbles [47]. For instance, at a concentration of 12 mg/L, Tween-80 can reduce the surface tension to 31.4 mN/m, while significantly enhancing the stability of the foam [48]. Ostwald maturation refers to the phenomenon where small bubbles gradually disappear and large bubbles gradually increase in size [48]. This is one of the main reasons for the instability of foam. Nonionic surfactants effectively inhibit Ostwald maturation by forming a stable interfacial film and increasing the electrostatic repulsion between bubbles. For instance, research has found that when Tween-80 is used in combination with Zn/FeO nanoparticles, the stability of the foam is significantly enhanced, and the foam lifespan is extended by 3 to 5 times [48].

#### 3.2.2. Bubble Stability Control

The stability of bubbles is crucial for their application in oil and gas fields. Micro-nano bubbles are prone to rupture due to surface tension, flow conditions, and chemical reactions, necessitating measures to enhance their stability. The stability of bubbles is effectively regulated through the addition of surfactants and the optimization of bubble generation techniques, which include various generation methods, as well as specific temperature and pressure conditions.

#### 3.2.3. Optimization of Bubble Injection Parameters

The effectiveness of micro-nano bubble injection is influenced by several parameters, including the injection rate, injection pressure, and injection method. Proper optimization of these parameters enables significant enhancement of oil displacement efficiency and improvement of resource recovery rates in oil and gas fields. The bubble injection rate should be adjusted according to the reservoir permeability and the stability of the bubbles to prevent excessive rates that could lead to bubble rupture or uneven distribution. The injection pressure should be regulated to ensure that bubbles effectively penetrate various reservoir layers, while preventing excessive pressure from compromising bubble stability. An appropriate injection method should be selected, such as continuous or intermittent injection, and adjustments should be optimized based on reservoir conditions to achieve a uniform distribution of bubbles and enhance the efficiency of oil displacement.

## 4. The Current Research Status

Extensive studies have demonstrated that MNBs are capable of persisting in bulk solutions for several months; however, the mechanisms underlying their stabilization require further refinement. These three fields cover critical stages of petroleum extraction. Drilling fluid optimization enhances drilling efficiency by improving the rheological properties and stability of the fluid. Enhanced oil recovery involves the injection of microbubbles to modify the behavior of reservoir fluids and increase hydrocarbon mobility, particularly in low-permeability and complex reservoirs. Oil–water separation utilizes microbubbles to improve separation efficiency, thereby facilitating wastewater treatment and resource recovery. As a gas–liquid dispersion system, micro-nano bubbles exhibit unique hydrodynamic characteristics. The three major application areas highlighted above demonstrate the comprehensive nature of micro-nano bubble technology in petroleum engineering, effectively addressing technical challenges encountered at various stages of oil extraction and showcasing its holistic technological profile within the field.

### 4.1. Application Status of MNBs in Petroleum Engineering Field

#### 4.1.1. Drilling Fluid Leakage Prevention and Plugging Technology Based on Gel Micro-Nano Microbubbles

During the drilling process, lost circulation significantly impacts drilling efficiency. MNB drilling fluid effectively prevents losses by reducing the hydrostatic pressure in the wellbore and forming a stagnant layer near the well wall [49]. Additionally, the coarse foam plugging technique intentionally allows drilling fluid to enter fractures, creating a “honeycomb”-like foam gel that generates “gas locking” and gel plugging in the loss channels, further enhancing the plugging effect [49]. Relevant researchers have found that the gel micro-nano foam drilling fluid has a better performance, higher gelation strength, and will not experience well leakage or stuck pipe.

#### 4.1.2. Drilling Fluid Performance Optimization

Drilling fluid, often referred to as the “blood of drilling”, encompasses a variety of circulating fluids that fulfill the diverse requirements of drilling operations through their multifaceted functions. The use of drilling fluids dates back nearly 150 years, with their primary types including water, mud, clay-free flushing fluids, emulsions, foam, and compressed air. However, as a circulating medium, the suspension capacity, pressure controllability, lubricity, cooling efficiency, and environmental impact of drilling fluids have become technical challenges. In recent years, the high specific surface area, prolonged retention time, excellent dispersibility and stability of MNBs, as well as their eco-friendly properties, have been confirmed. Incorporating these additives into drilling fluids to create a dispersed system presents numerous advantages, including enhancements in the rheological properties of the drilling fluid, improved chip suspension capability, increased thermal conductivity, and effective density adjustment [50,51].

The enhancement of the rheological properties of drilling fluid is crucial for optimizing drilling operations. MNBs significantly improve the rheological characteristics of drilling fluid, thereby increasing its adaptability to complex formation conditions encountered during drilling. Experimental data show that at 25 °C and a shear rate of 100 s^−1^, MNBs can reduce the apparent viscosity of drilling fluid by 15–20% [11,50,51]. The power-law fluid index n value was measured by a rheometer and increased from 0.6 to 0.8. Through the circular flow channel test, the efficiency of cuttings transport has been increased by 30–40%. Here, the n value represents the flow behavior index in the power-law model. *n* < 1 indicates shear thinning characteristics, and an increase in the value suggests that MNBs make the fluid behavior closer to that of Newtonian fluids (*n* = 1) while retaining the advantage of shear thinning. By reducing flow resistance and enhancing the shear-thinning properties of the liquid, MNBs improve the fluidity and rheological characteristics of drilling fluids [11]. Bubble suspension and drag reduction are critical functions of MNBs in drilling fluids, as they significantly enhance the suspension capacity of the fluid. Microbubbles enhance the volume of drilling fluid, thereby improving its capacity to suspend and transport cuttings. This improvement mitigates blockages that may arise from the accumulation of cuttings during the drilling process. Additionally, the introduction of bubbles effectively reduces flow resistance, aiding in improving drilling efficiency and lowering energy consumption during the process [51]. The density regulation of drilling fluid can be improved. MNBs serve to adjust the density of drilling fluid, particularly in scenarios where pressure and density variations need to be controlled during drilling. Through interaction with the liquid, MNBs facilitate precise density adjustments to ensure pressure equilibrium during drilling, thereby mitigating issues such as wellbore collapse [36]. Enhancing the contamination resistance of drilling fluids is critical for maintaining their effectiveness. The incorporation of MNBs into drilling fluids significantly aids in preventing the sedimentation and contamination of solid materials. These bubbles bind with solid particles to form stable bubble–solid complexes, which slow the settling rate of solids in the drilling fluid and enhance its stability [50,51].

MNBs have an extremely large specific surface area (SSA), as described by the formula $\;\mathrm{SSA}=\frac{6\sum n_{i}d_{i}^{2}}{\sum n_{i}d_{i}^{3}}$ (Equation (2)). This large SSA provides abundant adsorption sites for solid particles. According to the research of Etchepare R et al., the interaction between MNBs and solid particles at the gas–liquid interface can be enhanced through surface chemical effects, which is conducive to the formation of stable bubble–solid complexes [11]. High ζ potential of MNBs can effectively suppress the compression of the double electric layer, which not only enhances the stability of the foam but also promotes the combination of bubbles and solid particles [23,52]. The electrostatic repulsion or attraction caused by ζ potential helps to form a relatively stable structure of bubble–solid complexes. The internal high pressure of MNBs creates a pressure gradient around the bubbles. This pressure gradient can generate forces such as micro-convection, which increases the probability of collision between bubbles and solid particles, thereby promoting the formation of bubble–solid complexes [52].

Moreover, the chemical components found in traditional drilling fluids frequently pose significant environmental risks. In contrast, By reducing the number of additives from five to two key components, microfoam technology not only simplifies the formula of drilling fluid but also significantly lowers the usage of chemicals, with a reduction ratio of 40–60%. This improvement not only reduces the complexity of the drilling fluid but also lowers the carbon footprint during the production and transportation of chemicals. The life cycle assessment shows that the carbon footprint has been reduced by 30%. Research shows that through the response surface method and experimental optimization, the key components of microfoam drilling fluid include foaming agents, foam stabilizers and modified bentonite materials. For instance, in the optimal formula, the proportion of foaming agent is 3.98%, that of compound agent is 0.586%, that of stabilizer is 0.776%, that of sulfonated phenolic resin is 1%, and that of sodium carboxymethyl starch is 2% [17]. This formula not only enhances the performance of the drilling fluid but also strengthens its protective effect in soft coal seams, effectively preventing problems such as borehole collapse and stuck pipe [53].

Overall, the application of MNBs in drilling fluids presents several advantages, particularly in enhancing rheological properties, reducing drilling resistance, and improving thermal conductivity efficiency [54,55]. It is expected to become a key technology for boosting drilling efficiency and environmental performance in the future.

#### 4.1.3. Gel Micro-Nano Bubbles Enhance the Recovery of Horizontal Wells

**Figure 2:**
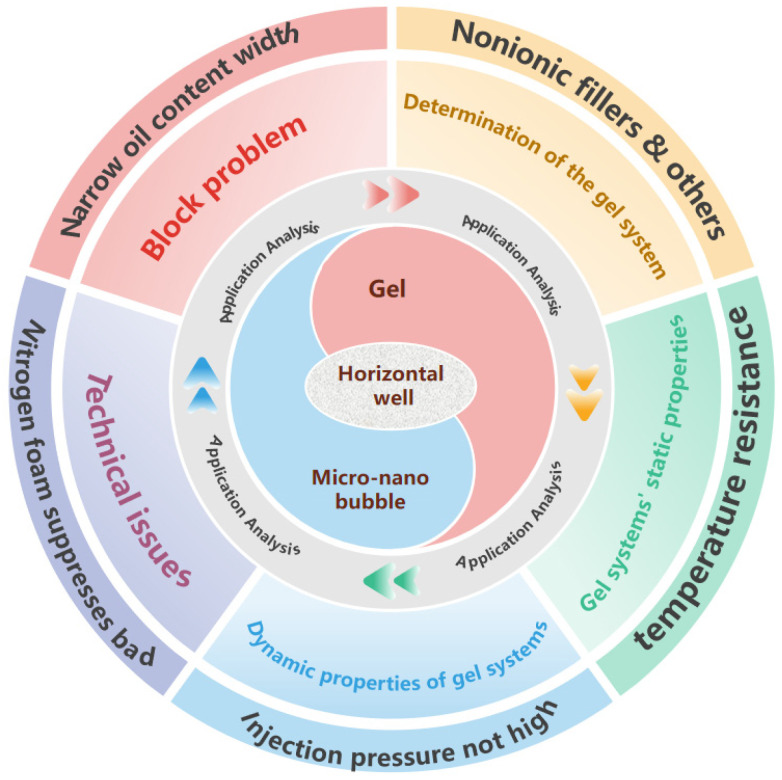
The application mechanism of gel micro-nano bubbles in horizontal well.

In horizontal well development, edge water breakthrough is a common issue. Microfoam gel technology, by optimizing the gel foam system, can effectively suppress edge water advancement, reduce the comprehensive water cut of the well cycle, and thereby improve development efficiency. This technology is not only simple to operate but also demonstrates significant water shutoff effects, offering a new solution for horizontal well development. Figure 2 shows the mechanism diagram of gel micro-nano bubbles applied in horizontal wells.

#### 4.1.4. Enhanced Oil Recovery

**Figure 3:**
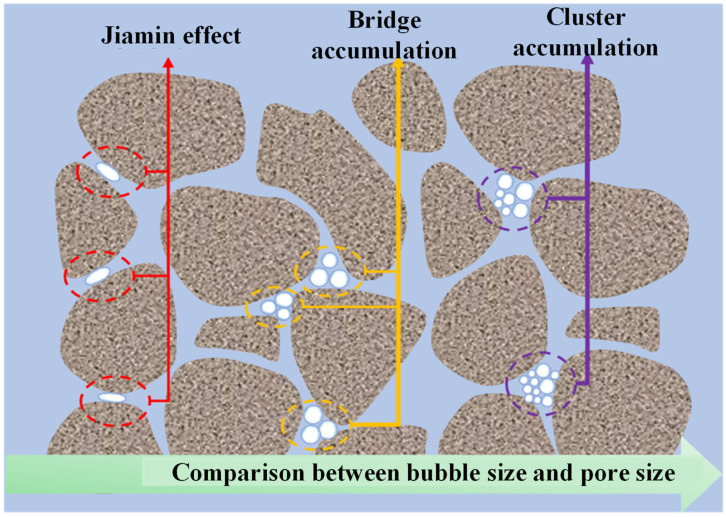
Correspondence between bubble size and pore size.

MNBs possesses substantial potential for enhancing oil recovery by improving extraction efficiency in low-permeability reservoirs, optimizing secondary and tertiary recovery processes, and minimizing chemical and energy consumption. Furthermore, they enhance the gas and water drive effects in oil and gas reservoirs. The enhancement of crude oil migration within microscopic pore throats is regarded as the primary mechanism through which micro-nano bubble technology improves recovery, particularly in marginal oilfields and inefficient reservoirs. The relationship between the size of MNBs and pore dimensions significantly influences bubble passability, thereby altering fluid flow patterns in heterogeneous pores [14], as illustrated in Figure 3. Specifically, it may be described as: First, when the diameter of the bubbles is smaller than that of the pore throats, microbubbles tend to influx in a disordered manner, resulting in “cluster accumulation” with the potential to lead to pore throat blockage [12]. Second, when the diameter of microbubbles is comparable to that of the pore throats, the passage of microbubbles through these pore throats may result in a phenomenon known as “bridging accumulation”, which tends to lead to temporary blockage [17]. Wu Binbin et al. noted that the accumulation of microbubbles bridging the intersections of vertical and horizontal microchannels leads to short-term blockage, which increases seepage resistance and alters the flow direction of subsequent fluids. When the diameter of microbubbles exceeds that of the pore throats, the additional pressure induced by the “Jamin effect” obstructs the passage of bubbles, resulting in blockages that compel fluids to alter their flow paths [14,53]. While the discussion may be accurate, whether the references cited are the earliest proposed warrants further investigation, as some authors may have referenced the research findings of others. Some research outcomes were neither the earliest proposed nor validated by alternative methods; in such instances, the earliest proposed outcomes should be utilized.

With the introduction of X-ray CT technology in the medical field, the hydrodynamic characteristics within pores have been confirmed. Zhao et al. utilized X-ray CT for visual analysis of core displacement experiments, with results indicating that CO_2_ microbubbles have the ability to delay finger development, thereby more efficiently penetrating areas with low porosity and low flow capacity [54]. X-ray computed tomography (CT) technology has significant advantages in studying the hydrodynamic characteristics within pores, but its application still has some limitations. X-ray CT research under laboratory conditions is usually conducted at the microscopic or mesoscopic scale, while the actual scale of oil fields involves a larger spatial range and more complex geological conditions. This scale difference may make it difficult to directly extrapolate the experimental results to the actual oilfield scenarios. For instance, the core samples used in laboratories are usually small in size and cannot fully reflect the complexity of the multi-scale pore structure in oil fields [55]. In addition, the fluid flow behavior under laboratory conditions may be affected by boundary effects, which is significantly different from the infinite boundary conditions in actual oil fields [56].

Li et al. also demonstrated through field tests that MNBs exhibit excellent plugging and profile control functions [57]. The results showed a 95.6% reduction in liquid production from high-pressure layers and a 53.6% increase in low-pressure layers, confirming the effective plugging of high-pressure layers by microbubbles [58]. In summary, MNBs combine enhanced oil recovery efficiency with environmental benefits, making them an innovative solution for improving the economic viability and sustainability of oil and gas extraction [12,58,59]. It should be noted that some literature refers to microbubbles without clearly distinguishing the concept of bubbles [51], whereas it actually denotes micron-sized bubbles. The key factor distinguishing the two lies in whether the bubbles exist in a dispersed form within the bulk solution.

Although field tests have demonstrated significant profile control effects (with a 95.6% reduction in liquid production in high-pressure layers), it should be noted that the geological conditions of different reservoirs, especially heterogeneity, can significantly affect the profile control results. For instance, in low-permeability fractured oil reservoirs, the water flooding effect is better in the fracture direction, while there may be no response on both sides of the fracture [60]. In addition, the vertical heterogeneity of the reservoir can also lead to uneven distribution of profile control agents in different layers, thereby affecting the overall effect [61]. At present, there are relatively few studies on the long-term stability of profile control agents. For instance, the stability of organic phenolic gel in high-temperature and high-salt environments may decline over time [62]. Therefore, when designing the MNBs’ profile control scheme, the heterogeneity characteristics of the reservoir need to be fully considered.

#### 4.1.5. Oil Water Separation

Oil–gas separation is an essential process in the petroleum industry, particularly during crude oil production, where efficient and cost-effective separation is crucial for enhancing productivity. Compared to macro bubbles, MNBs demonstrate superior adsorption capabilities when interacting with hydrophobic substances, resulting in exceptional treatment efficacy for small-sized and hard-to-degrade oil contaminants [57,63]. Currently, optimizing MNB generation technology, improving oil–water separation efficiency, and integrating techniques such as flocculation, synergistic air flotation, and chemical oxidation represent effective approaches to enhance separation performance [64]. On one hand, MNBs possess the ability to independently adsorb oil pollutants, thereby facilitating flotation. This mechanism enhances the collision and adhesion probability between oil droplets and bubbles, which significantly improves the separation efficiency of oil-in-water emulsions. (Xiaobing Li, 2016) presented a novel cyclonic micro-bubble flotation column (FCSMC) that achieved an oil removal rate of 92.19% [63], surpassing the performance of dissolved air flotation (DAF). On the other hand, MNBs also act as a bridge facilitating the interaction between macro bubbles and oil pollutants [11,65]. Furthermore, research examining the combination of MNBs with flocculation–air flotation has demonstrated that nano bubbles preferentially capture and adhere to flocculated oil droplets. This process results in the formation of aerated oily flocs, which ultimately assist MNBs in achieving effective flotation [40]. The technology of MNBs continues to encounter challenges, including high equipment costs and limited large-scale applications. However, with ongoing technological advancements, its potential applications in the treatment of oil-containing wastewater are significant.

### 4.2. Technical Comparison and Challenge Analysis at Home and Abroad

In the field of petroleum engineering, micro-nano bubble technology has emerged as an innovative technique for optimizing drilling fluids, attracting significant attention both domestically and internationally. There are notable differences in the application of this technology between domestic and international contexts. Table 3, Table 4 and Table 5 summarize the application approaches and case studies of micro-nano bubble technology across three major technical areas: optimization of drilling fluid performance, enhanced oil recovery, and oil–water separation. Additionally, a parallel analysis of the technical challenges encountered in these fields is conducted [36,66,67].

Under high-temperature and high-pressure conditions, the stability of microfoams significantly decreases, and one of the main reasons for this is the attenuation of the ζ potential. When the temperature exceeds 80 °C, the ζ potential of CO_2_ bubbles drops from 27 mV to 11 mV, causing the double electric layer (EDL) to compress, thereby reducing the stability of the foam [68]. The decrease in ζ potential will weaken the electrostatic repulsion between bubbles, promote the coalescence and rupture of bubbles, and ultimately lead to the instability of the foam. To optimize the foaming process and enhance the stability of the foam, this can be achieved by adjusting the chemical properties of the foaming agent. For instance, using amphiphilic Janus particles (such as NH_2_-SiO_2_-12C) as stabilizers can significantly enhance the stability of foam. This particle has an appropriate contact Angle (about 80°), a high ζ potential and good surface activity, which can effectively extend the drainage half-life of the foam from 448 s to 778 s under high-temperature and high-calcium conditions [68]. By optimizing the foaming process, such as using the membrane dispersion method, the ζ potential can be stabilized above ±20 mV, thereby effectively suppressing the compression of the double electric layer and enhancing the stability of the foam [68].

In the context of drilling fluid performance optimization applications, domestically, it is primarily utilized in conventional oil and gas fields. However, it exhibits relatively low technical stability and encounters challenges related to bubble collapse under high-temperature and -pressure conditions. Internationally, it is applied in extreme environments such as deepwater drilling, where bubble stability is better, but high costs and equipment complexity remain urgent challenges. In enhanced oil recovery applications, domestic technology is primarily utilized in low-permeability reservoirs, such as shale gas, where it has demonstrated effective results. However, its efficacy in high-viscosity reservoirs remains limited. On an international scale, micro-nano bubbles have been employed to enhance the mobility of oil and gas during secondary and tertiary recovery processes, yielding significant outcomes. Nonetheless, challenges such as uneven bubble size distribution and associated costs continue to pose obstacles. Regarding oil–water separation applications, domestic applications in wastewater treatment and conventional oil fields have shown good results, but stability in high-temperature environments is poor. Internationally, micro-nano bubble technology has been effectively applied in deepwater oil fields and the petrochemical industry, though controlling bubble generation remains challenging.

The application of micro-nano bubble technology in the field of petroleum engineering faces four major technical challenges: high stability, high adaptability, operability (including bubble surface modification, morphology regulation, and concentration control), and economic efficiency. To overcome these challenges, optimizing foaming processes and systems is essential for enhancing bubble generation efficiency and stability [69]. This optimization lays the foundation for the widespread application of micro-nano bubble technology in petroleum engineering, particularly in areas such as drilling, enhanced oil recovery, and oil–water separation.

The main differences between domestic and foreign technologies in bubble stabilization methods lie in the strategies for dealing with extreme conditions. In an aqueous solution system at 25–100 °C, the surface tension (γ) decreases by 40–60%, resulting in an exponential reduction in the internal pressure of bubbles (ΔP = 2γ/r) [70]. Foreign technology enhances bubble stability by optimizing the molecular structure of surfactants, such as using copolymers modified with polyethylene glycol (PEG), which can maintain a low surface tension at high temperatures [70]. Domestic technologies, on the other hand, rely more on adjusting the solution environment, such as reducing surface tension by adding inorganic salts (like NH_4_Cl and MgCl_2_), but this method has limited effect at extremely high temperatures [71]. In a high-temperature environment above 80 °C, thermal energy can destroy the monolayer of surfactants, increasing the probability of bubble coalescence by 3 to 5 orders of magnitude [72]. Foreign technologies have demonstrated excellent stability in deepwater applications by developing high-temperature resistant surfactants (such as siloxane-based surfactants) and mixed surfactant systems (such as MIBC-PEG) [73]. Domestic technology relies more on the addition of high-molecular polymers (such as xanthan gum and hyaluronic acid) to delay liquid discharge and bubble aggregation, but the stability under extreme high temperatures still needs to be further improved [74,75]. Therefore, domestic technologies can draw on international experience, combine molecular design and environmental regulation, and develop more efficient bubble stabilization methods.

Table 1 shows that the viscosity reduction effect (viscosity reduction of 35–40%) of domestic low-pressure bubble generators in shallow oil fields is consistent with the study of fuzzy spherical fluids conducted by Li et al. [50], but the stability drops sharply in the high-temperature environment of deep water [42]. The 15–22% resistance reduction rate of Halliburton’s high-voltage generator in the Gulf of Mexico reveals the correlation between bubble size and turbulence suppression [57], but the cost has restricted the promotion of this device.

## 5. Discussion

### 5.1. Applications of Functional MNBs

Functional micro-nano bubbles exhibit considerable promise in the field of petroleum engineering. They demonstrate significant potential for enhancing oil and gas recovery, optimizing water injection processes, and improving wastewater treatment efficacy [36,76,77]. Firstly, micro-nano bubbles enhance oil and gas recovery by reducing oil–water interfacial tension, improving fluid mobility, and facilitating bubble-driven oil displacement. Secondly, during oilfield water injection, micro-nano bubbles help increase water permeability and injection efficiency while improving fluid flow. Additionally, micro-nano bubbles play a significant role in oil–water separation and wastewater purification, effectively removing oil contaminants and treating wastewater. They are also combined with chemical flooding techniques, serving as drug carriers to enhance oil displacement effectiveness.

### 5.2. Targeted Rupture Technology of MNBs

The targeted rupture technology of micro-nano bubbles holds broad application prospects in the field of petroleum engineering [78,79]. This technology leverages the rupture characteristics of micro-nano bubbles under specific conditions to achieve precise control and optimization during oilfield extraction. Firstly, micro-nano bubbles rupture in a targeted manner within reservoirs, releasing energy that aids in unblocking clogged layers or fractures, thereby enhancing the permeability and fluidity of oil and gas [80]. This targeted rupture significantly enhances oil and gas recovery rates, proving especially beneficial in the development of complex and low-permeability reservoirs [80,81]. Secondly, the targeted rupture technology is integrated with other oil and gas extraction techniques, such as water flooding and chemical flooding, to optimize the extraction process [82]. During water flooding, the targeted rupture of micro-nano bubbles enhances water permeability, ensuring a more uniform injection and preventing water coning that may result from excessive injection [83]. Additionally, micro-nano bubbles function as carriers for chemical agents, enabling precise delivery to deeper reservoir layers and significantly enhancing oil displacement efficiency. However, the primary challenges of this technology lie in the precise control of bubble rupture timing and location, as well as in maintaining bubble stability under high-temperature and high-pressure reservoir conditions [82,83]. With the continuous advancement of technology, the targeted rupture technique utilizing micro-nano bubbles is anticipated to facilitate more efficient and environmentally friendly extraction of oil and gas within the field of petroleum engineering.

## 6. Conclusions and Foresight

MNBs exhibit unique physicochemical properties, including high stability, slow ascent, significant interfacial potential, and strong oxidative capabilities. With ongoing technological advancements and decreasing costs, MNBs are playing an increasingly significant role in enhancing oil and gas recovery, wastewater treatment, and equipment protection. Moreover, the high molecular weight and stable structure of the gel can make up for the shortcomings of micro-nano bubbles in MNBs technology, such as easy liquid separation and sedimentation, and improve the stability of the bubbles.

Applicative prospects or research directions for MNBs:

(1) MNBs significantly reduce oil–water interfacial tension, thereby facilitating the oil-water separation process.
(2) MNBs enhance the buoyancy of oil droplets, thereby improving the efficiency of oil-water separation.
(3) The combined application of MNB technology and gel can enhance the stability of drilling fluids.

Challenges and Solutions:

(1) Despite numerous laboratory validations and preliminary industrial applications, the large-scale implementation of MNBs technology in petroleum engineering continues to face significant challenges.
(2) Industrialization and cost control issues serve as the primary obstacles to the widespread adoption of MNBs.
(3) It is essential to establish a closer connection between the geological conditions of oil and gas reservoirs and bubble dynamics to gain a deeper understanding of bubble behavior and its effects under various conditions.
(4) The field application of Micro-Nano Bubbles (MNBs) should be advanced through enhanced technical validation and industrial testing.
(5) It is crucial to establish technical standards tailored to the specific needs of China’s oil and gas industry to promote the standardized and normative development of this technology.
(6) For gel micro-nano bubble technology, it is crucial to develop environmentally friendly chemical additives to reduce the potential environmental impact of gel microfoams.
(7) For technical optimization, developing low-energy-consumption generators and eco-friendly gel additives helps solve energy/environmental risks and fits oilfield on-site needs.

## 7. Methods

### 7.1. Terminology Definitions

As shown in Table 6, it is an explanation of some relevant professional terms in this article.

### 7.2. Key Equations

(1) Young–Laplace equation:

$$P_{\mathrm{in}}=P_{\mathrm{out}}+\frac{2\gamma }{r}$$

*P*_in_: Bubble internal pressure (unit: N/m^2^)

*P*_out_: Liquid-phase pressure (default 105 N/m^2^)

γ: Surface tension (default for water system: 72 mN/m)

r: Bubble radius

(2) Specific surface area formula:

$$\mathrm{SSA}=\frac{6\sum n_{i}d_{i}^{2}}{\sum n_{i}d_{i}^{3}}$$

Application limitations: Only applicable to non-interacting spherical bubbles. Corrections are required for CGA systems.

(3) Rising speed formula:

$$v_{b,\mathrm{vis}}=\frac{1}{f_{\mathrm{sc}}}\cdot \frac{g\rho_{L}d_{e}^{2}}{12\mu_{L}}$$

Scope of application: Viscous laminar flow (Reynolds number Re < 1).

## Figures and Tables

**Table 1:** Micro-nano bubble preparation technologies and their limitations.

Technology Name	Basic Principle	Technical Advantages	Limitations
Dissolved gas method	Differences in gas solubility in liquids under varying pressure conditions	High gas dissolution capacity, suitable for dissolving various types of gases	High equipment requirements and significant energy consumption
Ultrasonic method	Ultrasonic vibrations cause the formation, growth, and collapse of bubbles in liquids	Controllable bubble size, rapid gas dissolution	High equipment cost, localized effect variations
Electrolysis method	By electrolyzing liquids such as water, gas generated from the electrolytic reaction forms micro-nano bubbles	High gas purity, mature technology	High power consumption, limited bubble scale, single bubble form
Venturi method	Increased flow velocity in the pipeline reduces pressure, allowing gas to enter the liquid and form micro-nano bubbles	Simple operation, suitable for large-scale applications	Lower bubble generation efficiency, potential unevenness
Porous membrane technology	Uses porous materials to disperse gas flow in liquid, forming micro-nano bubbles	Capable of precise control over bubble size with good stability	Membrane clogging issues and cost problems
Airflow cutting method	Generates micro-nano bubbles through shear interaction between highspeed airflow and liquid	Simple production and rapid bubble generation	Poor bubble stability, potential unevenness, and high energy consumption
Jet Method	Liquid is sprayed through high-pressure airflow to specific orifices or nozzles, forming high-velocity bubble streams	Rapid bubble generation, easy operation	Uneven bubble distribution, poor bubble stability

**Table 2:** Taxonomy of bubble systems in petroleum engineering.

Classification	Size Range	Interfacial Structure	ζ-Potential (Typical)	Generation Methods	Petroleum Use Cases
Micro-Nano Bubbles (MNBs)	50 nm–50 μm	Surfactant monolayer	−30 to −50 mV (25 °C)	Ultrasonic/Venturi	EOR, shale gas mobilization
Colloidal Gas Aphrons (CGAs)	10–100 μm	Surfactant bilayer + polymer	−15 to −25 mV (80 °C)	High-shear mixing	Drilling fluid weighting
Microfoams	100–500 μm	Protein/polymer composite	−5 to −10 mV (60 °C)	Mechanical frothing	Pipeline drag reduction

**Table 3:** Comparison and challenge analysis of MNBs at home and abroad in drilling fluid performance optimization.

Technical Field	Domestic Technology	Foreign Technology	Technical Bottleneck
Drilling fluid performance optimization	*Bubble injection drilling fluid*: Improve rheology and chip-carrying ability through low-pressure bubble generators. *Low temperature conventional environment*: Suitable for conventional drilling operations and optimizing drilling fluid performance. *Application cases*: In China, micro-nano bubbles are typically introduced into drilling fluids through low-pressure bubble generators to enhance their rheological properties and cuttings-carrying capacity, thereby improving drilling efficiency. In several relatively shallow oil and gas fields, the application of bubble technology has successfully reduced mud viscosity and improved the stability of drilling operations.	*High pressure bubble generator and bubble stabilizer*: The performance of drilling fluid in high-temperature and high-pressure environments is optimized by generating stable micro-nano bubbles at high pressure. *Deepwater drilling operations*: Especially suitable for deep water and unconventional oil and gas drilling. *Application cases*: Halliburton used high-pressure bubble generators in Gulf of Mexico deep-water drilling. The drag reduction rate of drilling fluid can be increased to 15–22% by generating 200 nm bubbles. bubble generators boosting fluid’s chip-carrying/fluidity, speeding drilling to stabilize efficiency.	*Poor bubble stability*: Because micro-nano bubble technology is prone to rupture in high-temperature and high-pressure environments, the performance of the drilling fluid to lose sustained and stable. *Limited deep-layer applications*: The technology is mostly concentrated in shallow oil and gas extraction, lacking application cases in high-temperature and high-pressure environments.

**Table 4:** Comparison and challenge analysis of MNBs at home and abroad in oil–water separation.

Technical Field	Domestic Technology	Foreign Technology	Technical Bottleneck
Oil–water separation	*Oil–water separation enhancer*: Micro-nano bubbles improve oil–water separation effect and are suitable for low-temperature conditions. *Wastewater treatment*: Improve oil–water separation efficiency. *Application cases*: A petrochemical company used micro-nano bubble technology to treat wastewater, which successfully improved the efficiency of oil–water separation. By injecting micro-nano bubbles into the wastewater, the oil–water interface tension is effectively improved, and the oil droplets quickly gather, reducing the separation time and cost. In addition, this technology has also achieved remarkable results in oil–water separation in conventional oil fields, especially in low-temperature environments.	*Bubble flotation and aggregation technology*: Improves oil–water separation efficiency and is suitable for deep-water oil fields. *Industrial wastewater treatment*: Improves the effect of oil–water separation and widely used in the petrochemical industry. *Application cases*: Shell used micro-nano bubble flotation technology to perform oil-water separation in its deep-water oil fields, successfully improving the efficiency of oil–water separation. Micro-nano bubbles not only improve the aggregation effect of oil droplets, but also optimize the separation time of the oil–water interface and reduce wastewater discharge. This technology has been widely used in many deep-water oil fields.	*Poor bubble stability*: Micro-nano bubbles exhibit low stability in water, potentially leading to inconsistent separation performance. *Difficulty in bubble size control*: It is difficult to accurately control the size and injection amount of bubbles, affecting the oil–water separation effect.

**Table 5:** Comparison and challenge analysis of MNBs at home and abroad in improving the recovery efficiency.

Technical Field	Domestic Technology	Foreign Technology	Technical Bottleneck
Improving the recovery efficiency	*Micro-nano bubble flooding*: Used for shale gas/oil extraction to enhance oil and gas flowability. *Increase production by water injection*: Improve water injection operation efficiency. *Application cases*: In a Chinese shale gas field, micro-nano bubble oil-driving technology enhances oil-gas mobility and recovery by reducing reservoir viscosity, optimizing oil–water interaction, and improving water injection permeability, with good results in low-permeability reservoirs.	*Secondary/tertiary oil recovery*: Enhanced oil recovery rate, suitable for high-temperature and high-pressure oil fields. *Bubble enhanced injection technology*: Used to improve recovery, especially in high-viscosity reservoirs. *Application cases*: Chevron applies micro-nano bubble technology to U.S. shale oil field secondary recovery, reducing viscosity, boosting oil–gas flow, recovery rates and field economic benefits by cutting water injection frequency.	*Uneven bubble generation*: Under complex reservoir conditions, the non-uniform generation and distribution of micro-nano bubbles may hinder the enhancement of recovery rates. *Limited effect*: In some high-viscosity reservoirs, the improvement in recovery rates from micro-nano bubbles is relatively modest.

**Table 6:** Explanations of professional terms and sources of literature.

Term/Symbol	Definition	Origin Reference
*MNBs*	Micro-nano bubbles (Diameter range: 50 nm–50 μm)	[11,32]
*Jamin effect*	The flow resistance effect generated by bubbles at the throat of the hole (applicable to bubbles > 10 μm)	[14]
*ζ potential*	Zeta potential, characterizing the stability of surface charge of bubbles (critical value: ±30 mV)	[11]
*SSA*	The Specific Surface Area is calculated according to Equation (2)	[1]
*f_sc_*	Correction factor for surface contaminants inhibiting the internal circulation of bubbles (range 1.2–1.5)	[24]

## Data Availability

No new data were created or analyzed in this study. Data sharing is not applicable to this article.
